# Comparison of Various Frequency Matching Schemes for Geometric Correction of Geostationary Ocean Color Imager

**DOI:** 10.3390/s19245564

**Published:** 2019-12-16

**Authors:** Jong-Hwan Son, Han-Gyeol Kim, Hee-Jeong Han, Taejung Kim

**Affiliations:** 1 Department of Geoinformatic Engineering, Inha University, 100 Inharo, Michuhol-Gu, Incheon 22212, Korea; json8520@inha.edu; 2 3DLabs Co. Ltd., 56, Songdogwahak-ro, Yeonsu-gu, Incheon 21984, Korea; khanai@3dlabs.co.kr; 3 Korea Ocean Satellite Center, KIOST, 385, Haeyang-ro, Yeongdo-gu, Busan 49111, Korea; han77@kiost.ac.kr

**Keywords:** frequency matching, GOCI, geometric correction, phase correlation, gradient correlation, orientation correlation

## Abstract

Current precise geometric correction of Geostationary Ocean Color Imager (GOCI) image slots is performed by shoreline matching. However, it is troublesome to handle slots with few or no shorelines, or slots covered by clouds. Geometric correction by frequency matching has been proposed to handle these slots. In this paper, we further extend previous research on frequency matching by comparing the performance of three frequency domain matching methods: phase correlation, gradient correlation, and orientation correlation. We compared the performance of each matching technique in terms of match success rate and geometric accuracy. We concluded that the three frequency domain matching method with peak search range limits was comparable to geometric correction performance with shoreline matching. The proposed method handles translation only, and assumes that rotation has been corrected. We need to do further work on how to handle rotation by frequency matching.

## 1. Introduction

The Geostationary Ocean Color Imager (GOCI) is a payload of the Communication, Ocean and Meteorological Satellite (COMS) launched in 2010 [1]. It performs ocean observation missions of a 2500 km × 2500 km area over the Korean Peninsula, Japan, eastern China, and northern Taiwan. The GOCI acquires data eight times a day from noon to 7 o’clock Coordinated Universal Time (UTC) [2].

**Figure 1:**
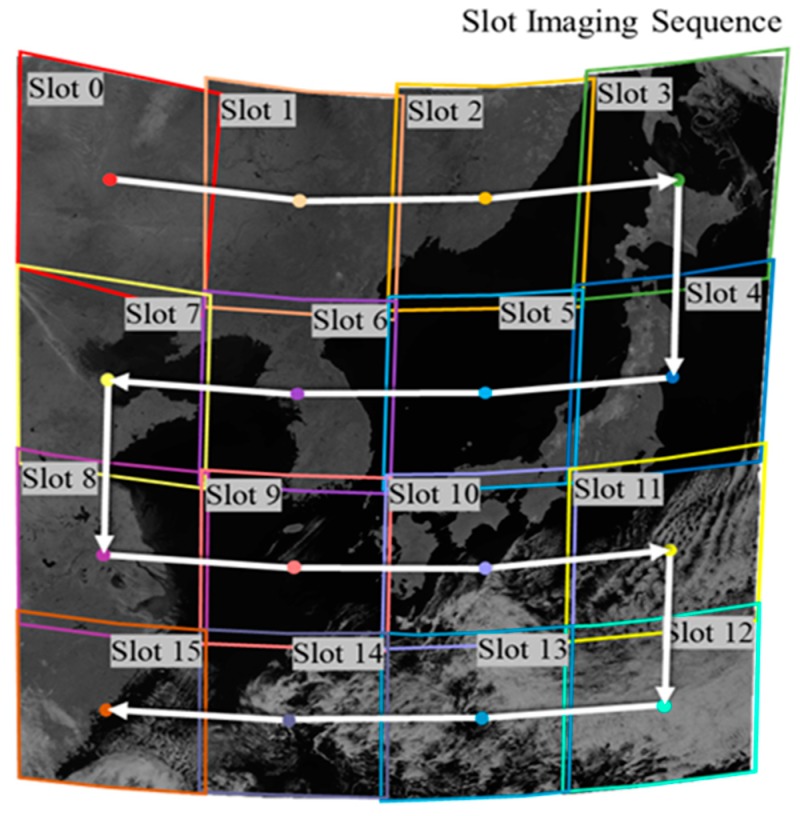
Geostationary Ocean Color Imager (GOCI) slot arrangement and imaging sequence (Reprinted from [4]).

GOCI image data are composed of 16 consecutive slots acquired by a sensor that scans the observation area in x and y directions (see Figure 1). It is necessary to perform precise geometric corrections and image mosaicking of the 16 slots to make one GOCI Level 1B image. Currently, precise geometric correction is performed by shoreline matching. Ground control point (GCP) chips, small image segments over shorelines and precise geographic coordinates at the center of the segments, are prepared in advance. GOCI slots are matched against GCP chips to estimate the precise orientation of the image slots. Using the estimated orientations, geometric distortions of image slots due to the Earth’s curvature are removed. Image slots after geometric correction are stitched together to make one full 1B scene [3].

However, not all GOCI slots have sufficient shorelines. For example, slots 0 and 1 in Figure 1 are inland slots, and slots 12, 13, and 14 are over an ocean area without shorelines. It is very difficult to apply shoreline matching over these slots and to robustly estimate the precise geolocation of the image slots. To handle such slots, we proposed a frequency matching scheme, and showed that adjacent slots can be matched by applying frequency matching to the overlapping areas of two slots [5,6]. We have further shown that frequency matching can be combined with shoreline matching to make one full 1B scene [7]. From shoreline matching, the absolute orientation of image slots was estimated and from frequency matching, the relative orientation of slots without shorelines. By combining the two matching results, it was possible to mosaic all 16 slots with improved geometric accuracy.

In this paper, we aimed to expand our previous research in order to further improve the frequency matching performance in terms of accuracy and robustness. While our previous findings were based on phase correlation matching, we here considered two additional frequency matching schemes: gradient correlation [8] and orientation correlation matching [9]. We compared the performance of the three frequency matching schemes and proposed an optimal strategy for GOCI geometric correction.

In Section 2, we briefly reviewed the principle of frequency domain matching and the differences among phase correlation, gradient correlation, and orientation correlation matching. Section 3 describes the experiment dataset and test methods. Section 4 reports the experiment results, and proposes an optimal frequency matching strategy for GOCI geometric correction. Section 5 concludes this paper with its findings and limitations, and suggests further research issues. In 2020, the Korean government plans to launch a GOCI-II, which is a successor payload of GOCI. We performed this study to develop the geometric correction method of GOCI-II.

## 2. Frequency Domain Matching

Since an image is a sampled dataset in two-dimensional (2D) space, 2D discrete Fourier transformation (DFT) can be used to convert it into the frequency domain (Equation (1)):

(1) F(k,l)=DFT(f(x,y)) ,

where f(*x,y*) is a 2D image, and F(*k,l*) is its frequency spectrum. As a result of the transformation, each pixel value of the spectrum is obtained as a complex function (Equation (2)). From the complex frequency spectrum, we can obtain the magnitude, |F(*k,l*)|, and phase, ∅(*k,l*), of the corresponding spatial frequency signal (Equations (3) and (4)):

(2) F(k,l)= R(k,l)+jI(k,l),

(3) $$\;\left|F\left(k,l\right)\right|=\sqrt{R^{2}\left(k,l\right)+I^{2}\left(k,l\right)},\;$$

(4) $$\emptyset \left(k,l\right)=\tan^{-1}\left[\frac{I\left(k,l\right)}{R\left(k,l\right)}\right]\;,\;$$

The spectrum can be transformed back to the original image using inverse discrete Fourier transformation (IDFT). By manipulating the spectrum, and performing an inverse discrete Fourier transform, the original image can be sharpened, blurred, or emphasized [10]. 

**Figure 2:**
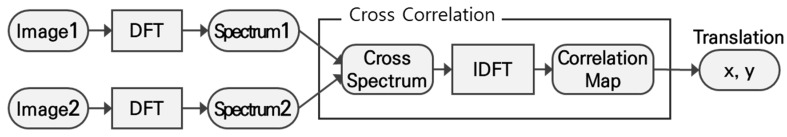
Frequency domain matching process.

Frequency domain matching is conducted by analyzing the correlation between two spectrums in the frequency domain. The overall matching process is shown in Figure 2. We transformed two images into spectrums of the frequency domain using DFT. Then, we generated a correlation map between the two spectrums by multiplying them and applying IDFT. Position (x, y) of a peak point with the largest value in the correlation map indicates the amount of translation between the two images [8,9,11,12].

Depending on how we generate frequency spectrums and how we multiply the two spectrums, we can design different frequency matching schemes. Phase correlation (PC) is a matching technique performed by cross-correlation analysis between spectrums (F and G) transformed from the original images (f and g) [9,11]. The cross-correlation of the PC is performed as shown in Equation (5). Note that G* is a complex conjugate of spectrum G. In Equation (5), the magnitude of the spatial frequency signal is eliminated, and consequently, matching is performed using only the phase values [11,13].

(5) $$\mathrm{PC}=\mathrm{IFFT}\left(\frac{F\left(k,l\right)G^{*}\left(k,l\right)}{\left|F\left(k,l\right)G^{*}\left(k,l\right)\right|}\right),\;\mathrm{where},\;F\left(k,l\right)=\;\mathrm{DFT}\left(f\left(x,y\right)\right)\;G\left(k,l\right)=\;\mathrm{DFT}\left(g\left(x,y\right)\right)$$

Gradient correlation (GC) is a matching technique that performs cross correlation over Fourier spectrums transformed from the gradient matrix of the original image [8]. As shown in Equation (6), the gradients in the x and y directions were set as real and imaginary parts of the original signal. The gradient matrix (fg, gg) was transformed into the Fourier spectrums (Fg, Gg) through discrete Fourier transform, and cross-correlation analysis is performed, as shown in Equations (6) and (7):

(6) $$f_{g}\left(x,y\right)=\;\frac{\partial f\left(x,y\right)}{\partial x}+\;i\frac{\partial f\left(x,y\right)}{\partial y}$$

$$\;g_{g}\left(x,y\right)=\;\frac{\partial g\left(x,y\right)}{\partial x}+\;i\frac{\partial g\left(x,y\right)}{\partial y}\;,\;$$

(7) $$\;\mathrm{GC}=\mathrm{IFFT}\left(F_{g}\left(k,l\right)G_{g}{}^{*}\left(k,l\right)\right)\;,\;\mathrm{where},{\;F}_{g}\left(k,l\right)=\;\mathrm{DFT}\left(f_{g}\left(x,y\right)\right)\;\;\;\;\;\;\;\;\;\;\;\;\;\;\;\;\;\;\;\;\;G_{g}\left(k,l\right)=\;\mathrm{DFT}\left(g_{g}\left(x,y\right)\right)$$

GC was reported to provide better matching performance, particularly if signals in the original images are not strong [7,8,9,13]. 

Orientation correlation (OC) performs a Fourier transform from the signum function of the gradient matrix (Equation (8)) and performs cross-correlation. The signum function converts the gradient matrix to an orientation matrix, having only the orientation information of the image gradients [12]:

(8) $$f_{o}\left(x,y\right)=\;\mathrm{sgn}\left(\frac{\partial f\left(x,y\right)}{\partial x}+\;i\frac{\partial f\left(x,y\right)}{\partial y}\right)\;\;g_{o}\left(x,y\right)=\;\mathrm{sgn}\left(\frac{\partial g\left(x,y\right)}{\partial x}+\;i\frac{\partial g\left(x,y\right)}{\partial y}\right)\;,\;\mathrm{where},\;\mathrm{sgn}\left(x\right)=\;\{\begin{array}{ll} 0 & if\;\;\left|x\right|=0\\ \frac{x}{\left|x\right|} & otherwise \end{array}$$

Cross-correlation analysis of the OC is performed as seen in Equation (9):

(9) $$\mathrm{OC}=\mathrm{IFFT}\left(F_{o}\left(k,l\right)G_{o}{}^{*}\left(k,l\right)\right)\;,$$

$$\;\mathrm{where},{\;F}_{o}\left(k,l\right)=\;\mathrm{DFT}\left(f_{o}\left(x,y\right)\right)\;\;G_{o}\left(k,l\right)=\;\mathrm{DFT}\left(g_{o}\left(x,y\right)\right)$$

## 3. Data and Test Method

### 3.1. Experiment Data

**Figure 3:**
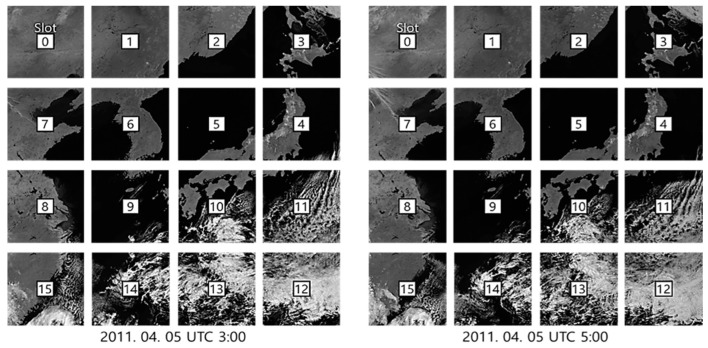
GOCI Level 1A band 8 data.

For the experiments, we used eight GOCI Level 1A datasets obtained from noon to 7 o’clock on 2011-04-05 UTC. Note that the GOCI Level 1A data came with radiometric correction and without geometric correction. GOCI Level 1A consists of 16 slots and each slot has 1413 X 1430 pixels. The GOCI can acquire images in eight bands (six visible bands, two near-infrared bands). The GSD (ground sample distance) of GOCI at the center of target area is 500 m. Since GOCI can perform acquiring and transferring 16 slots in less than 30 min [1], the time intervals between slots was less than 1.875 min. In this paper, we used the near infrared No. 8 band, which has an advantage in matching because clouds, soil, and ocean regions are clearly distinguished (Figure 3).

**Figure 4:**
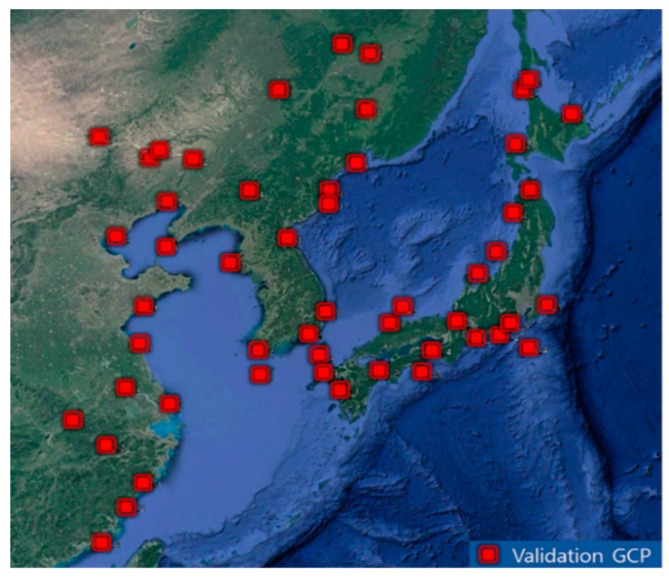
Validation points distribution (reprinted from [4]).

To analyze the accuracy of various frequency matching schemes, a total of 52 validation points were generated using Google Earth by identifying features and recording their geographic coordinates. Figure 4 shows the distribution of validation points. Since slots 12, 13, and 14 were occluded entirely by clouds, no validation points were obtained for these slots. For other slots, four validation points were generated per slot. It is notable that, although we aimed to apply frequency matching to handle slots without shorelines, we collected validation points from the slots with shorelines for quantitative accuracy estimation. For the slots without shorelines, we show the performance of frequency matching through visual inspection. The reported positional accuracies of Google Earth [14] are much smaller than the spatial resolution of GOCI images. We expect that validation points obtained in this way are sufficient to check the accuracy of our matching scheme.

### 3.2. Test Methods

**Figure 5:**
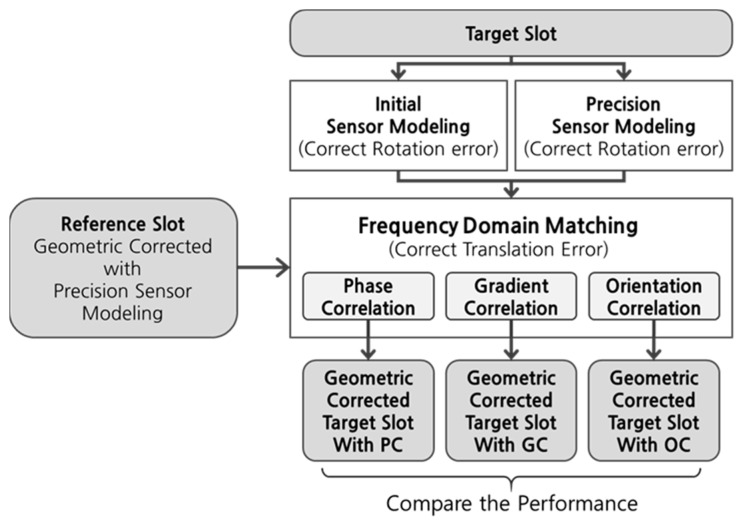
Overall test procedures.

In this paper, we applied frequency matching between a reference slot, to which precision sensor modeling has already been applied using shoreline matching, and a target slot, which needs geometric correction. To accomplish this, we assumed that transformation between the reference slot and the target slot is a rigid transformation, and that the rotation error of the target slot can almost be eliminated using the initial sensor modeling. Figure 5 shows the overall test procedures. We first applied precision sensor modeling to reference slots [4]. Next, we applied initial sensor modeling to target slots using initial ephemeris data provided by the satellite. We then applied frequency matching between the reference slot and target slot. Furthermore, to validate our assumption that rotation error of the target slot could almost be eliminated using initial sensor modeling, we applied precision sensor modeling to target slots and applied frequency matching between the reference slot and target slot. In this step, we conducted frequency matching schemes based on PC, GC, and OC, respectively, and compared their performance using the validation points. 

The matching proposed here is to handle a target slot without shorelines or without GCPs. Slot 12~14 are such target slots. However, the check the accuracy of the frequency matching performance, we needed to use slots with shorelines or validation points. Therefore, for the experiments, we used Slots 0~11 and Slot 15.

**Figure 6:**
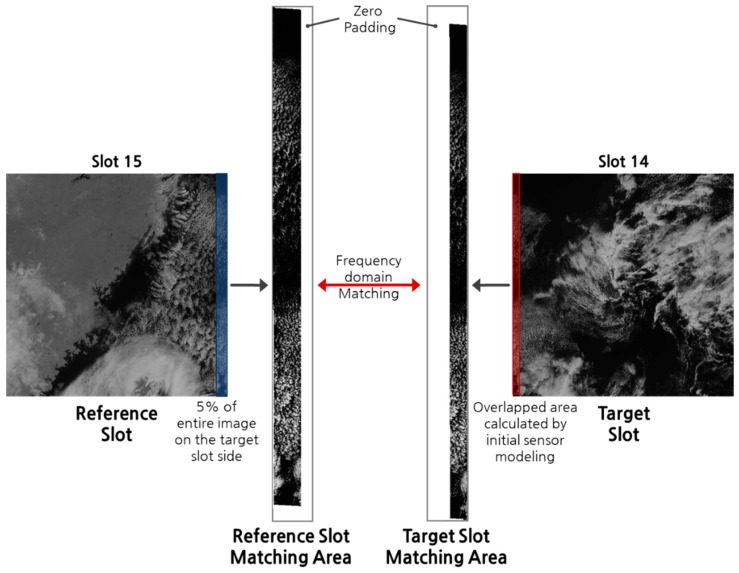
Example of setting the matching area (2011.04.05 UTC 03:00 Slot 14,15).

For a valid performance comparison, it is important to select an appropriate matching area between the reference and target slots. In performing frequency domain matching, the closer the matching area is to a real overlapped area, the greater the matching success rate [5]. Therefore, the matching area needs to be precisely defined. We projected the reference and target slots to an object space with their initial sensor models and set an overlapped area between the projected slot coverages as the matching area of the target slot. For robust frequency matching, the matching area of the reference slot must include the matching areas of the target slot. According to the GOCI slot acquisition scheme, the overlap between adjacent slots does not exceed 5% of the image slot size. Therefore, we set 5% of the reference slot as the matching area of the reference slot. The matching areas defined in this way will have image sizes that are different from each other. We made the size of the reference and target matching areas identical by using zero-padding (Figure 6).

After matching the area setting, the three frequency matching schemes were applied, and the amount of translation between the reference and target slots was estimated. The precise orientation of the target slots was determined by applying the translation to the initial orientation of the slots. The accuracy of the precise orientation of the target slots was analyzed from the validation points for the target slots.

**Figure 7:**
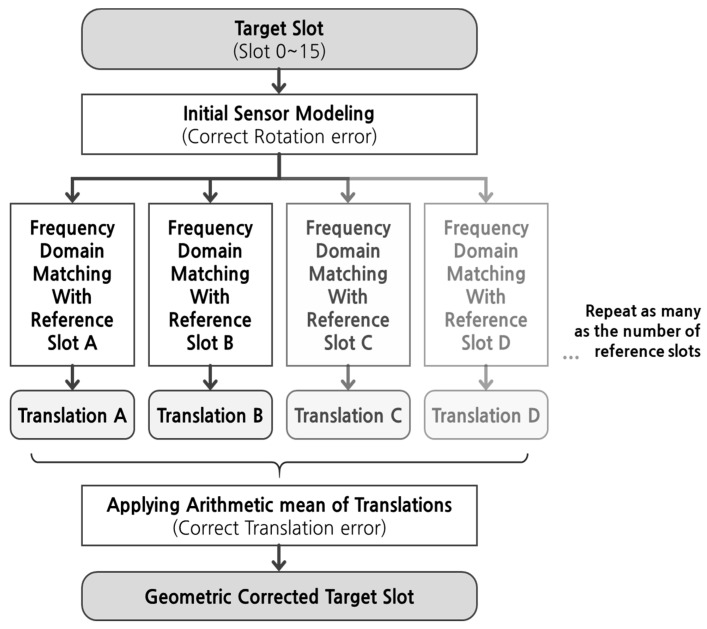
Final geometric correction process using frequency domain matching.

Depending on the location of a target slot, we could apply frequency domain matching and estimate translation up to four times (with left, right, up, and down reference slot). In order to generate the GOCI Level 1B image, which performed precise geometric corrections and image mosaicking of the 16 slots, we needed to determine the final translation. As shown in Figure 7, we determined the final translation as an arithmetic mean of the translations. By applying the final translation, we determined the final precise orientation of each slot and made one GOCI Level 1B image. Finally, we compared the accuracy of Level 1B generated by frequency domain matching and precision sensor modeling.

## 4. Results and Discussion

In this paper, three experiments were carried out to analyze the performance of frequency matching. In the first experiment, the frequency domain matching success rate was analyzed from the three frequency domain matching techniques and the search range setting in the correlation map. In the second experiment, we performed precise geometric correction using frequency domain matching between each slot pair of GOCI data, and analyzed the performance. In the last experiment, we implemented geometric correction based on the best frequency domain matching method and compared the performance with the geometric correction with shoreline matching.

### 4.1. Analysis of Frequency Matching Success Rate

We applied the PC, GC, and OC frequency matching techniques to the GOCI Level 1A data and analyzed the matching success rate. For one set of GOCI Level 1A data, frequency matching was applied to 36 reference-target pairs. Since we used eight GOCI Level 1A data, frequency matching was performed on a total of 288 pairs. We analyzed the matching success rates through a comparison with true translations obtained manually. If an error in a matching result was more than three pixels, we determined it as an outlier.

We applied the three frequency matching by two difference peak search ranges. First, we searched for a peak value within the entire region of the correlation map. Second, we set the initial peak position from the initial sensor models of the reference and target slots, and limited the search range. According to the nominal accuracy of the ephemeris data, we determined the magnitude of the search radius as six pixels.

As shown in Table 1, we found that the success rate was significantly improved in all matching techniques by setting the search range as the constant radius in the correlation map.

When we set the search range as the entire correlation map, OC showed the best matching success rate. On the other hand, when the search range was set as a constant radius, all matching techniques (PC, GC, OC) showed a high matching success rate. 

**Figure 8:**
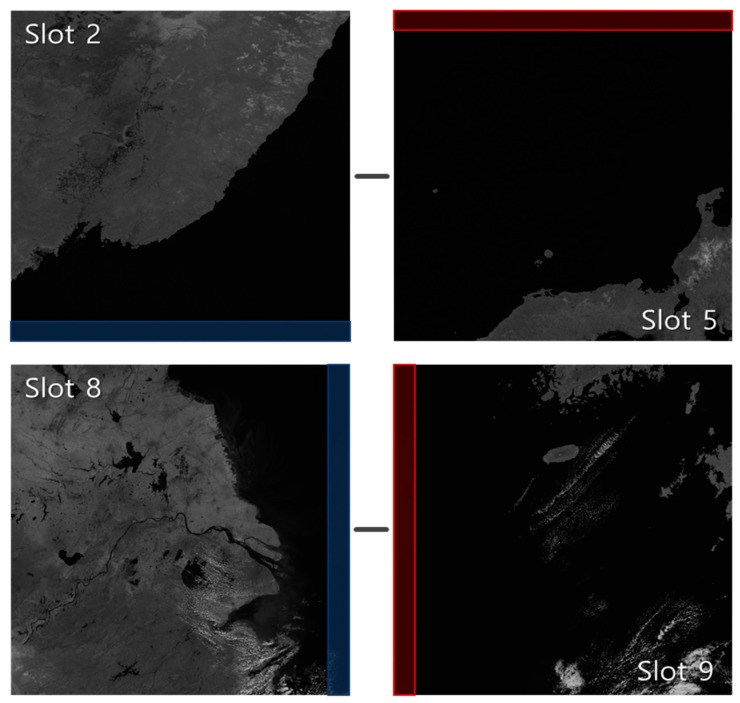
Example of Pair A (2011.04.05 Coordinated Universal Time (UTC) 03:00 slots 2–5, slots 8–9).

Table 2 shows the frequency matching success rate by slot. The results in this table were obtained by limiting the peak search radius. Table 2 shows that mismatches occurred mostly between specific reference-slot pairs (pair A) such as slots 2–5 and slots 8–9. We found that all these image pairs had an ocean area greater than 95% within the matching areas (see Figure 8). Although we developed frequency matching to handle slots without shorelines, the decrease in the matching success rate over ocean areas was inevitable. Note that one can apply automated outlier detection and removal. In this paper, we focused on the comparison of the frequency matching performance on this harsh environment. In particular, the slot 2–5 pair showed the worst performance among pair A. Since this pair not only had matching areas with an ocean area of greater than 95%, but also a longer time interval than other pairs in the pair A group. Whereas slots in other pair A were acquired consecutively, slot 5 was the third acquired slot after slot 2 acquisition. As more time intervals increased, more changes occurred at ocean textures, and the success rate of the frequency domain matching decreased. When there are clouds with movement, texture change would be more significant and affect the performance of frequency matching. This issue will remain as our future research.

**Figure 9:**
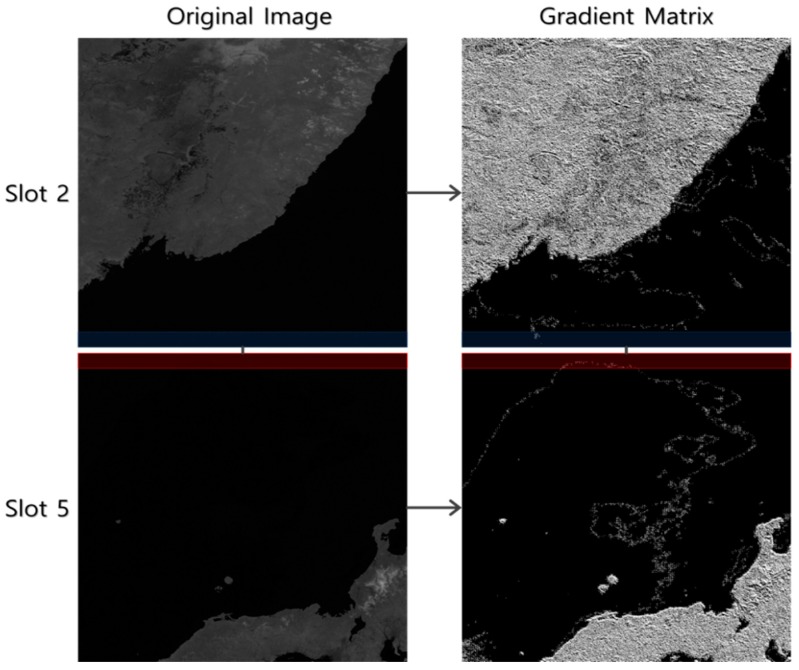
Example of the Pair A gradient matrix (2011.04.05 UTC 03:00 Slots 2–5).

When we compared the performance among the three schemes, OC performed more robustly over the ocean areas. An ocean area has very dark brightness values in band 8(NIR), and the gradient change rate is not large. It seems that if the image gradient over this area is used for matching, the influence of noise on the texture information is strengthened (Figure 9). With orientation correlation, only the orientation of the image gradient was used, and the influence of noise on the texture information was reduced.

### 4.2. Analysis of Geometric Correction Accuracy of Frequency Matching

We compared the accuracy of geometric correction by the frequency matching techniques. The accuracy was analyzed by the root mean square difference between the pixel location calculated from the estimated orientation and the true pixel location of the validation points (Table 3).

When setting the search range as the entire correlation map, the performance was better in the following order: PC, GC, and OC. On the other hand, in geometric correction with the search range, the performance was better in the following order: OC, GC, and PC. However, the performance of the three matching techniques was almost similar.

With OC, despite having the best matching success rate, it showed the lowest geometric correction performance when executed with no search range setting. It seems that OC commits the most serious gross errors when an outlier occurs. However, if we prevent the occurrence of outliers by setting the search range in the correlation map, the most precise matching is possible with OC. On the other hand, PC has a relatively small amount of error when an outlier occurs, but has low precision.

In Table 4, we compared the accuracy of the three frequency domain matching techniques using target slots corrected by initial sensor models and by precision sensor models. We performed this comparison to check the validity of using initial sensor models for correcting target the rotation errors of the slots. We also performed this comparison to check whether frequency matching introduces additional errors to precision sensor models. In the table, ISM represents the initial sensor model errors of the target slots. We corrected the target slots by the initial sensor models and applied the frequency domain matching techniques between the reference slot and target slot. The accuracy of each technique (ISM2PC, ISM2GC, ISM2OC) was checked using validation points. Furthermore, we applied precision sensor modeling to target slots and the accuracy of the precision sensor models was checked using validation points (PSM). We then applied frequency domain matching between the reference slots and the target slots corrected by PSM. We checked the accuracy of each frequency matching (PSM2PC, PSM2GC, PSM2OC) using the validation point.

In all experiments, the reference slots were corrected by precision sensor models. The results of frequency domain matching in Table 4 were obtained by limiting the peak search radius. In every slot, the geometric error of the initial sensor modeling (ISM) was reduced by using frequency domain matching, ISM2PC, ISM2GC, and ISM2OC. Their errors were larger than the cases with the target slots corrected by PSM or by PSM2PC, PSM2GC, and PSM2OC. However, the differences were not very big, indicating that the frequency matching scheme proposed here could be used for slots without shorelines and that correcting target slots by initial sensor models could be sufficient for applying frequency matching.

We can see that the accuracy of frequency matching using targets corrected by the precision sensor models, PSM2PC, PSM2GC, and PSM2OC, was very close to that of PSM. This means that the increase in error due to frequency domain matching was not significant. This also indicates the applicability of frequency matching to slots without shorelines. As previously mentioned, most of the outliers were among slots with an ocean area greater than 95% within their matching areas. As a result, the geometric correction accuracy was also lower than with other pairs. The large errors between slots 1–0 and 7–0 in ISM2PC, ISM2GC, and ISM2OC were because slot 0 was an inland slot, and shoreline matching was not performed accurately.

We generated GOCI Level 1B using all frequency matching results. We set all reference slots that paired with one target slot and applied the initial sensor modeling of the target slot and precision sensor modeling of reference slots. Then, we performed frequency domain matching with each reference slot. Finally, we determined the final translation as an arithmetic mean of translations. In Table 5, we generated GOCI Level 1B using the final translation and compared it with those by precision sensor modeling with GCP. Performance of geometric correction with three frequency domain matching was improved when compared with using only one frequency domain matching. Since we used all frequency matching results and determined the final translation as an arithmetic mean of translations, the effects of each reference slot’s geometric error and frequency domain matching error were reduced. The average accuracy was 2.0 pixels from PC, 2.0 pixels from GC, and 1.9 pixels from OC, while it was 2.2 pixels from precision sensor modeling. In most slots, geometric correction with frequency domain matching (PC, GC, OC) showed performance close to geometric correction with shoreline matching. It is notable that slot 0 had a small number of, and a bad distribution of, GCPs, and geometric correction with frequency domain matching showed better performance than shoreline matching.

**Figure 10:**
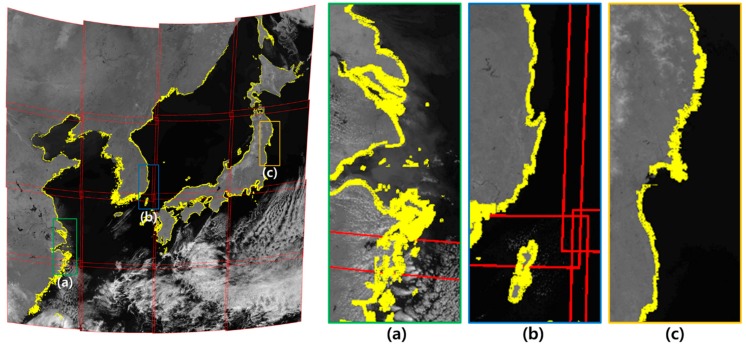
Level 1B image generated by Orientation Correlation (OC) frequency domain matching. ((**a**)–(**c**) are the magnified images of some region).

Figure 10 shows a mosaic image generated by performing geometric correction with OC. The yellow shoreline is a visual representation of the actual shoreline. The actual shoreline and the shoreline of a mosaic image generated by geometric correction were almost the same.

**Figure 11:**
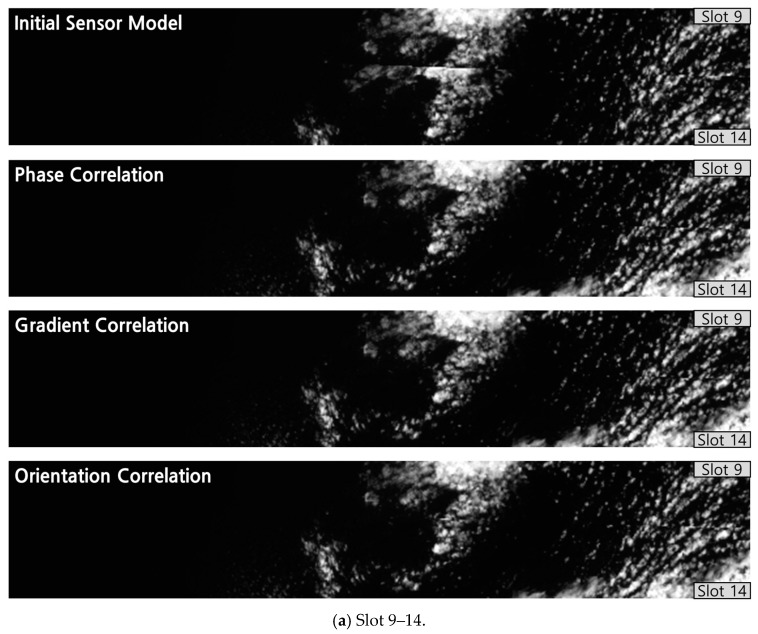

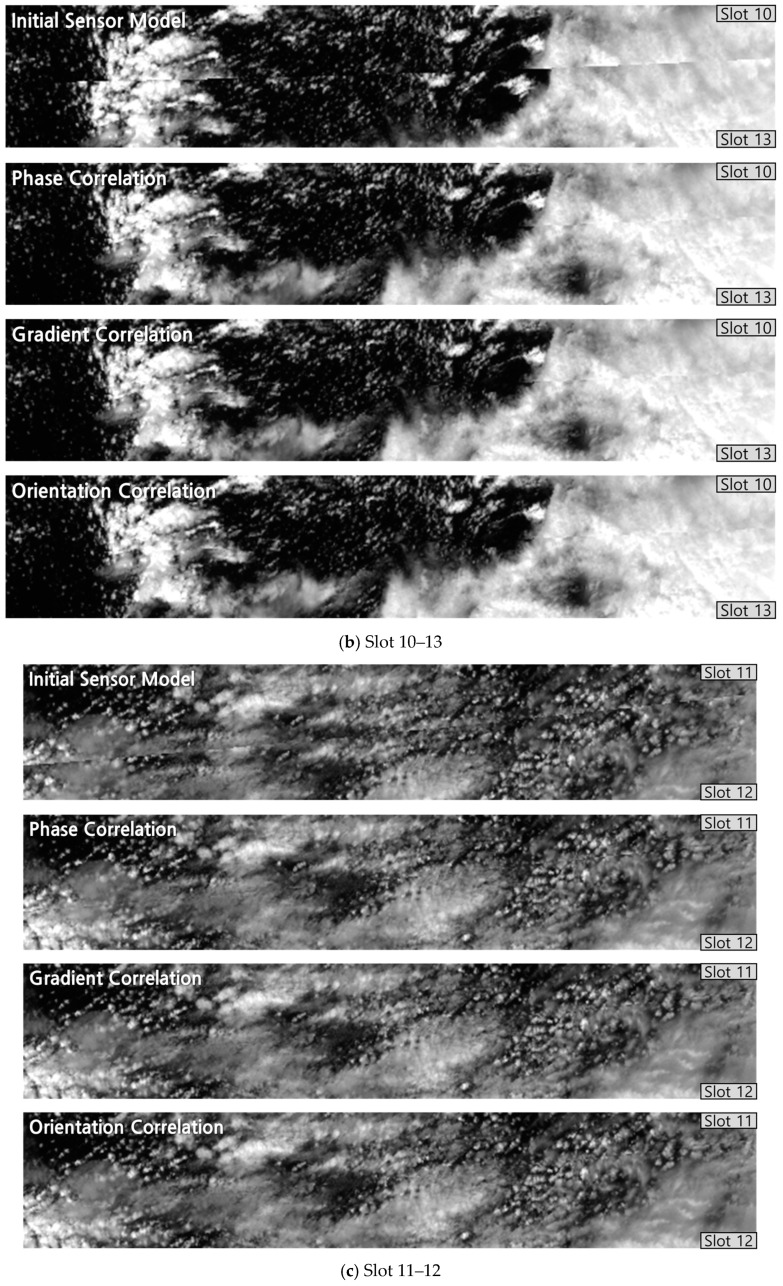
Comparison of seam lines between slots in each mosaic image (2011.04.05 UTC 03:00).

In order to analyze the accuracy of geometric correction for slots 12, 13, and 14, where there were no validation points, we visually analyzed the seam line between slots in the mosaic image (Figure 11). We found that the seamline between slots was hardly ever identified in the mosaic image using frequency domain matching. The geometric correction results from all frequency domain matching method (PC, GC, OC) showed the good performance in the seamline.

## 5. Conclusions

In this paper, we proposed a precision geometric correction method of GOCI data in order to handle images without shorelines. We proposed three frequency domain matching techniques (phase correlation, gradient correlation, and orientation correlation) and analyzed their matching performances. We concluded that the proposed frequency domain matching method (PC, GC, OC) with peak search range limits showed a close performance to precision sensor modeling. When the peak search range was not limited, both the matching success rate and geometric correction performance were lower in all matching methods. OC commits the most serious gross errors when an outlier occurs. However, if we are able to remove the outliers, the most precise matching is possible with OC. On the other hand, PC has a relatively small amount of error when an outlier occurs, but precision is low. The proposed method only handles translation, and assumes that rotation has been corrected. In future research, we will study how to perform frequency domain matching multiple times within one pair by dividing the matching area and will apply frequency matching to correct rotation errors.

## Figures and Tables

**Table 1:** Matching success rates according to the matching techniques and search range settings.

Search Range	Phase Correlation	Gradient Correlation	Orientation Correlation
	No. of Inliers/ No. of Outliers (Success Rate)	No. of Inliers/ No. of Outliers (Success Rate)	No. of Inliers/ No. of Outliers (Success Rate)
**Entire** **Correlation Map**	213/75 (73.96%)	247/41 (85.76%)	250/38 (86.81%)
**Constant** **Search Radius**	271/17 (94.10%)	273/15 (94.79%)	276/12 (95.83%)

**Table 2:** Matching success rate by slot.

TargetSlot	Ref.Slot	Phase Correlation		Gradient Correlation		Orientation Correlation	
		No. of Inliers	No. of Outliers	No. of Inliers	No. of Outliers	No. of Inliers	No. of Outliers
0	1	8	0	8	0	8	0
	7	7	1	8	0	8	0
1	0	8	0	8	0	8	0
	2	6	2	7	1	8	0
	6	8	0	8	0	8	0
2	1	8	0	8	0	8	0
	3	8	0	8	0	8	0
	5	5	3	4	4	5	3
3	2	7	1	8	0	8	0
	4	8	0	8	0	8	0
4	3	8	0	8	0	8	0
	5	8	0	8	0	8	0
	11	7	1	8	0	8	0
5	2	2	6	1	7	3	5
	4	8	0	8	0	8	0
	6	8	0	8	0	7	1
	10	8	0	8	0	8	0
6	1	8	0	8	0	8	0
	5	8	0	8	0	7	1
	7	8	0	8	0	8	0
	9	8	0	8	0	8	0
7	0	7	1	8	0	8	0
	6	8	0	8	0	8	0
	8	8	0	8	0	8	0
8	7	8	0	8	0	8	0
	9	6	2	5	3	6	2
	15	7	1	8	0	8	0
9	6	8	0	8	0	8	0
	8	7	1	8	0	8	0
	10	8	0	8	0	8	0
10	5	8	0	8	0	8	0
	9	8	0	8	0	8	0
	11	8	0	8	0	8	0
11	4	8	0	8	0	8	0
	10	8	0	8	0	8	0
15	8	8	0	8	0	8	0
A. Ocean Area > 95%		51	13	50	14	52	12
B. Otherwise		218	6	223	1	224	0
**Total**		269	19	273	15	276	12

(■ : A pair with more than 95% of the ocean area in the matching area).

**Table 3:** Accuracy of geometric correction according to matching technique.

Search Range	Phase Correlation (Only Inliers/ Only outliers)	Gradient Correlation (Only Inliers/ Only outliers)	Orientation Correlation (Only Inliers/ Only outliers)
**Entire** **Correlation** **Map**	16.6 pixels (2.6 pixels/56.4 pixels)	20.4 pixels (2.5 pixels/64.2 pixels)	23.4 pixels (2.4 pixels/80.8 pixels)
**Constant** **Search Radius**	3.0 pixels (2.8 pixels/7.8 pixels)	2.9 pixels (2.6 pixels/7.3 pixels)	2.8 pixels (2.5 pixels/7.6 pixels)

**Table 4:** Accuracy of frequency matching based on initial models and precision models.

Target Slot	Ref. Slot	ISM	ISM2PC	ISM2GC	ISM2OC	PSM	PSM2PC	PSM2GC	PSM2OC
		(Pixels)	(Pixels)	(Pixels)	(Pixels)	(Pixels)	(Pixels)	(Pixels)	(Pixels)
0	1	37.6	2.7	2.6	2.7	5.4	5.3	4.6	4.7
	7		3.4	2.5	2.5		5.4	5.0	5.0
1	0	34.7	4.8	4.9	5.0	2.4	2.5	2.9	2.4
	2		3.6	2.8	3.8		2.4	2.7	2.7
	6		1.8	1.8	1.8		2.4	1.9	2.1
2	1	37.3	3.2	3.0	2.9	2.2	2.1	1.8	1.8
	3		3.3	3.2	3.1		3.2	3.3	3.3
	5		5.7	5.8	5.3		2.1	3.1	3.1
3	2	34.8	4.7	4.5	4.5	1.8	1.8	2.4	2.4
	4		2.0	1.6	1.6		1.8	2.7	2.7
4	3	29.9	2.8	2.6	2.7	2.0	2.0	1.2	1.2
	5		1.9	1.9	1.9		2.4	2.6	2.6
	11		3.4	2.5	2.2		2.0	2.2	2.0
5	2	28.2	6.0	7.0	6.8	1.6	1.6	2.3	2.3
	4		1.5	1.5	1.5		1.3	1.2	1.2
	6		2.9	2.3	3.3		1.8	2.0	2.0
	10		1.4	1.4	1.3		1.6	1.6	1.6
6	1	30.4	3.7	3.4	3.9	1.4	1.3	1.4	1.8
	5		2.5	2.9	3.3		1.8	2.0	2.0
	7		3.0	2.7	2.8		1.3	1.2	1.0
	9		2.0	2.0	2.0		1.4	1.5	1.5
7	0	33.0	5.5	5.2	4.3	1.0	1.0	2.1	2.2
	6		1.7	1.7	1.8		1.2	1.3	1.5
	8		3.0	3.0	3.1		1.0	2.0	2.0
8	7	30.1	1.9	1.8	1.8	2.2	2.2	1.9	1.9
	9		4.6	4.9	4.8		2.8	3.1	3.4
	15		3.2	2.2	1.9		2.2	1.4	1.3
9	6	27.8	1.9	2.0	2.0	1.8	1.7	1.6	1.5
	8		3.1	3.3	3.0		3.3	4.0	3.9
	10		3.3	3.3	1.2		1.8	1.2	1.7
10	5	26.6	2.1	1.9	1.9	2.0	2.0	2.0	2.0
	9		1.7	1.8	1.8		2.9	3.2	2.3
	11		3.1	2.9	2.8		0.9	0.9	0.9
11	4	26.2	2.4	1.8	1.4	2.0	2.0	2.1	2.0
	10		2.4	2.4	2.4		3.7	3.7	3.7
15	8	28.2	2.5	2.3	2.4	1.8	1.8	2.2	2.2
A. Ocean Area > 95%		-	4.1	4.2	4.0	-	2.3	2.8	2.8
B. Otherwise		-	2.7	2.5	2.4	-	2.1	2.1	2.1
**Total**		31.1	3.0	2.9	2.8	2.2	2.2	2.3	2.3

(■: A pair with an ocean area greater than 95% in the matching area).

**Table 5:** Accuracy of Level 1B.

Tar. Slot	Ref. Slot	Frequency Domain Matching (pixels)			Precision Sensor Modeling (pixels)
		PC	GC	OC	
0	1,7	2.0	1.6	1.6	5.4
1	0,2,6	2.6	2.7	2.9	2.4
2	1,3,5	2.7	2.8	2.6	2.2
3	2,4	1.9	1.9	1.9	1.8
4	3,5,11	2.0	1.8	1.6	2.0
5	2,4,6,10	2.1	2.1	2.0	1.6
6	1,5,7,9	1.7	1.7	1.6	1.4
7	0,6,8	2.9	2.8	2.5	1.0
8	7,9,15	2.0	2.0	1.8	2.2
9	6,8,10	1.2	1.2	1.1	1.8
10	5,9,11	0.9	0.8	0.7	2.0
11	4,10	2.3	2.0	1.8	2.0
15	8	2.5	2.3	2.4	1.8
**Average**		**2.0**	**2.0**	**1.9**	**2.2**
